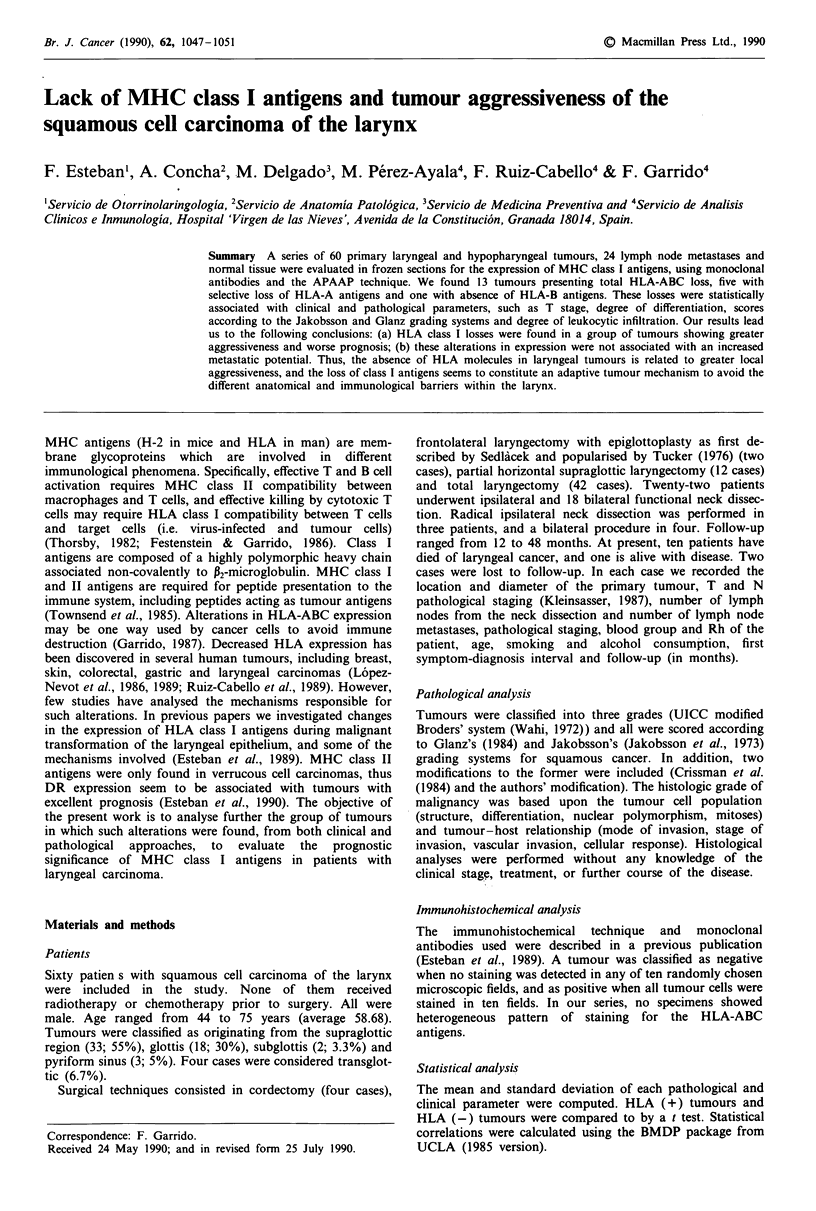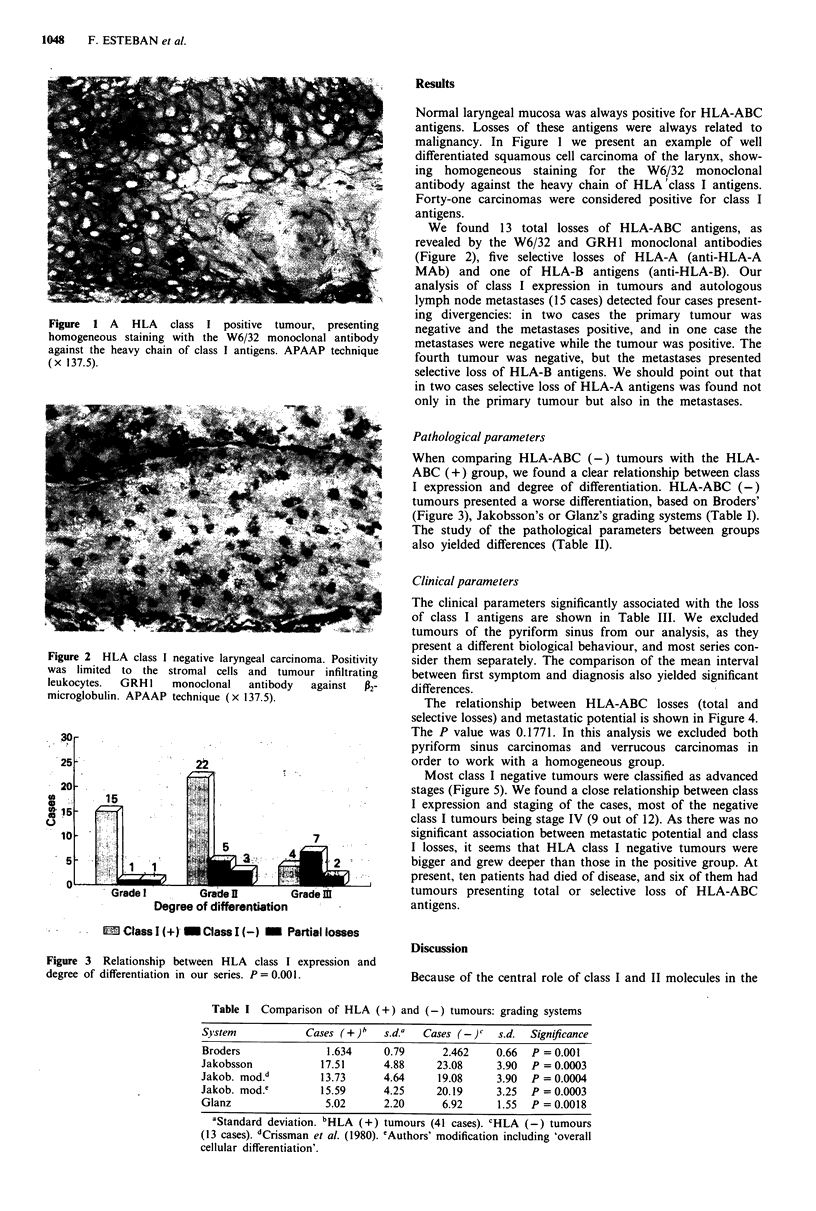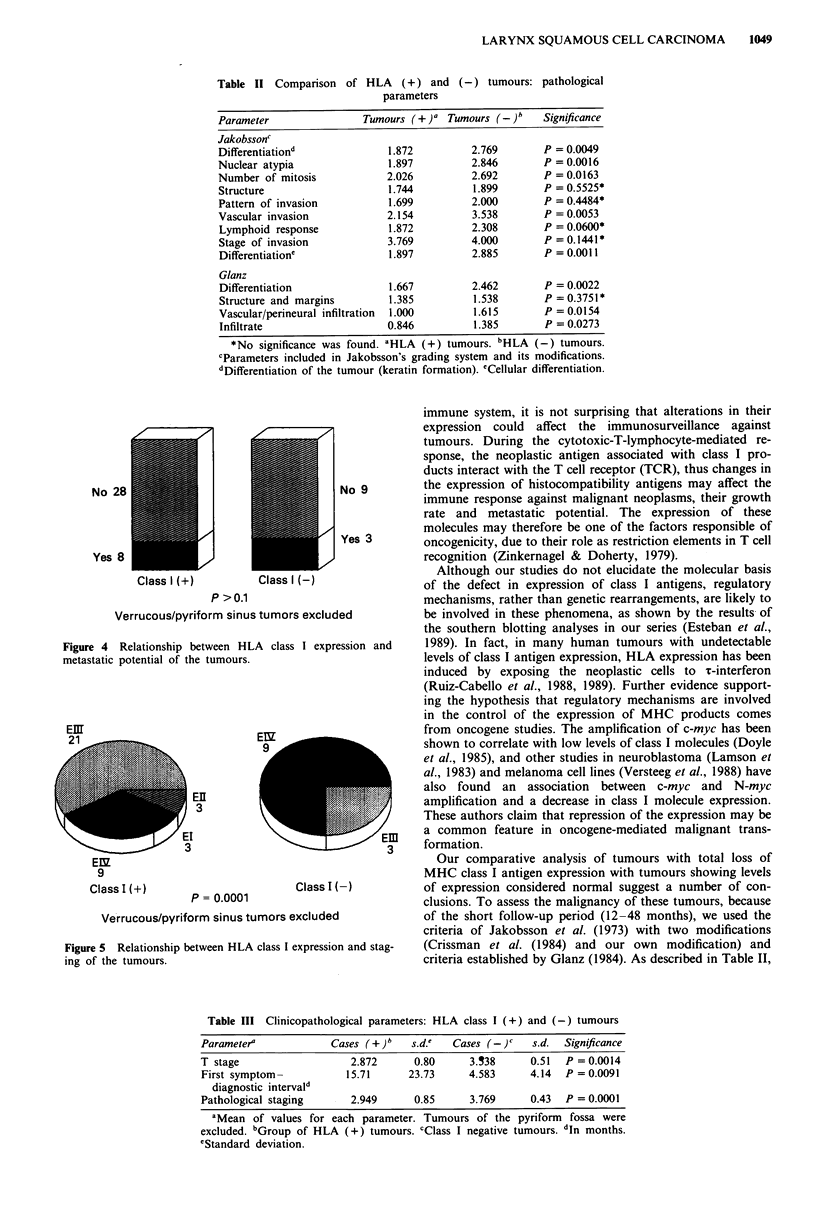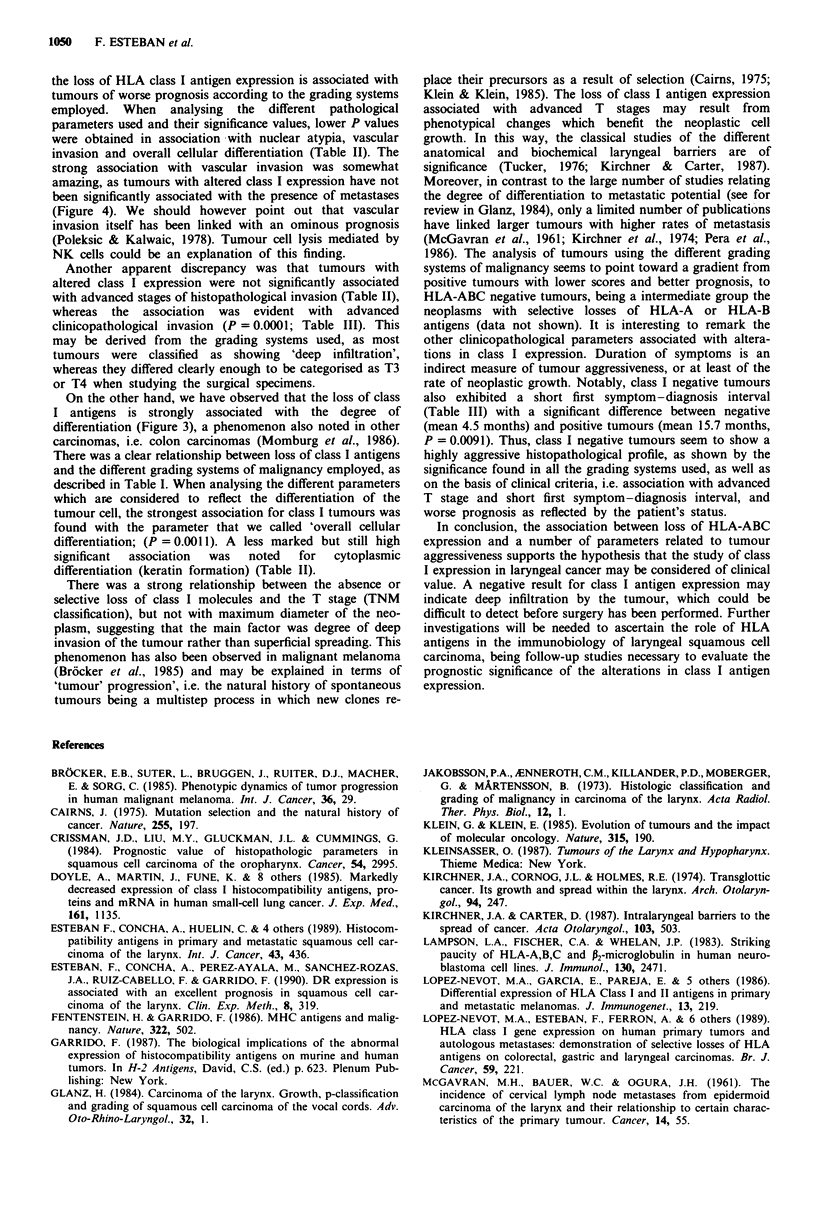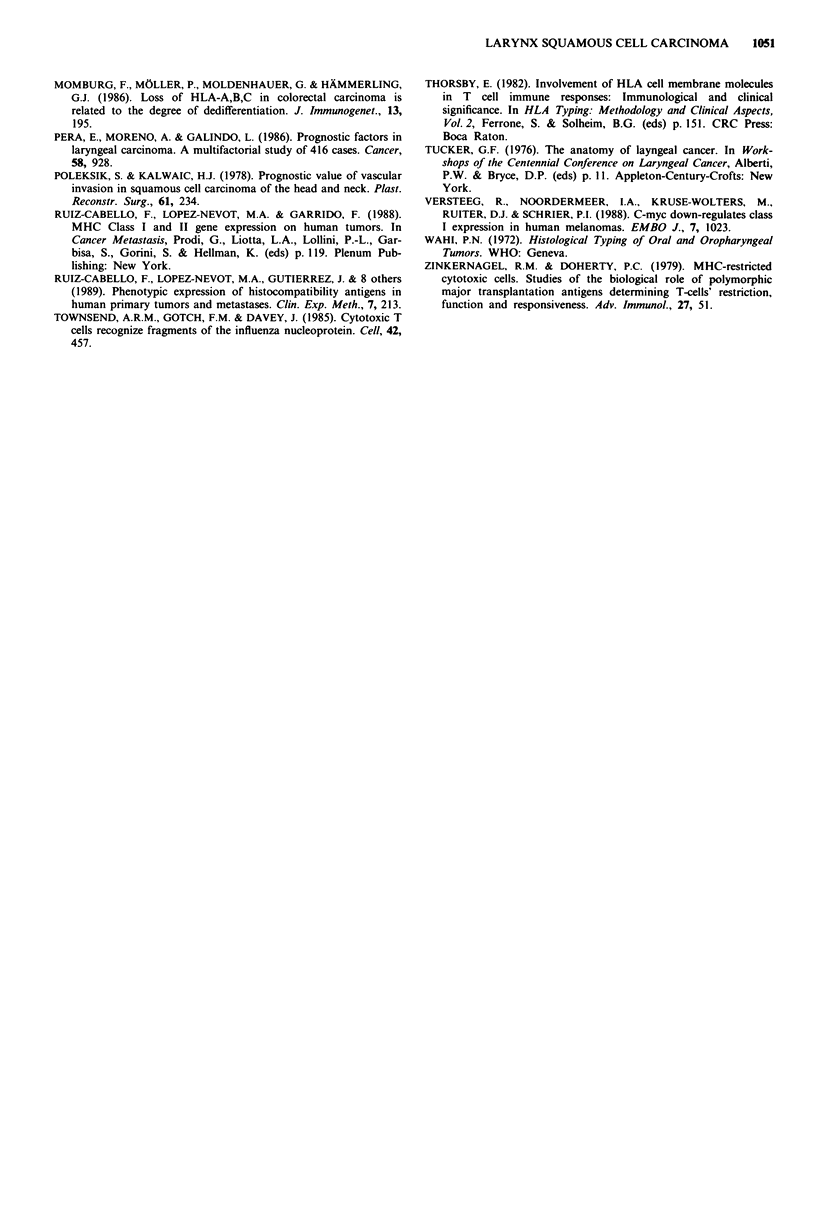# Lack of MHC class I antigens and tumour aggressiveness of the squamous cell carcinoma of the larynx.

**DOI:** 10.1038/bjc.1990.437

**Published:** 1990-12

**Authors:** F. Esteban, A. Concha, M. Delgado, M. Pérez-Ayala, F. Ruiz-Cabello, F. Garrido

**Affiliations:** Servicio de Otorrinolaringología, Hospital Virgen de las Nieves, Granada, Spain.

## Abstract

**Images:**


*Br. J. Cancer* (1990), **62**, 1047–1051 © Macmillan Press Ltd., 1990

### Lack of MHC class I antigens and tumour aggressiveness of the squamous cell carcinoma of the larynx

F. Esteban[1], A. Concha[2], M. Delgado[3], M. Pérez-Ayala[4], F. Ruiz-Cabello[4] & F. Garrido[4]

[1]*Servicio de Otorrinolaringología,* [2]*Servicio de Anatomía Patológica,* [3]*Servicio de Medicina Preventiva and* [4]*Servicio de Analisis Clínicos e Inmunología, Hospital 'Virgen de las Nieves', Avenida de la Constitución, Granada 18014, Spain.*

**Summary** A series of 60 primary laryngeal and hypopharyngeal tumours, 24 lymph node metastases and normal tissue were evaluated in frozen sections for the expression of MHC class I antigens, using monoclonal antibodies and the APAAP technique. We found 13 tumours presenting total HLA-ABC loss, five with selective loss of HLA-A antigens and one with absence of HLA-B antigens. These losses were statistically associated with clinical and pathological parameters, such as T stage, degree of differentiation, scores according to the Jakobsson and Glanz grading systems and degree of leukocytic infiltration. Our results lead us to the following conclusions: (a) HLA class I losses were found in a group of tumours showing greater aggressiveness and worse prognosis; (b) these alterations in expression were not associated with an increased metastatic potential. Thus, the absence of HLA molecules in laryngeal tumours is related to greater local aggressiveness, and the loss of class I antigens seems to constitute an adaptive tumour mechanism to avoid the different anatomical and immunological barriers within the larynx.

MHC antigens (H-2 in mice and HLA in man) are membrane glycoproteins which are involved in different immunological phenomena. Specifically, effective T and B cell activation requires MHC class II compatibility between macrophages and T cells, and effective killing by cytotoxic T cells may require HLA class I compatibility between T cells and target cells (i.e. virus-infected and tumour cells) (Thorsby, 1982; Festenstein & Garrido, 1986). Class I antigens are composed of a highly polymorphic heavy chain associated non-covalently to $\beta_2$-microglobulin. MHC class I and II antigens are required for peptide presentation to the immune system, including peptides acting as tumour antigens (Townsend *et al.*, 1985). Alterations in HLA-ABC expression may be one way used by cancer cells to avoid immune destruction (Garrido, 1987). Decreased HLA expression has been discovered in several human tumours, including breast, skin, colorectal, gastric and laryngeal carcinomas (López-Nevot *et al.*, 1986, 1989; Ruiz-Cabello *et al.*, 1989). However, few studies have analysed the mechanisms responsible for such alterations. In previous papers we investigated changes in the expression of HLA class I antigens during malignant transformation of the laryngeal epithelium, and some of the mechanisms involved (Esteban *et al.*, 1989). MHC class II antigens were only found in verrucous cell carcinomas, thus DR expression seem to be associated with tumours with excellent prognosis (Esteban *et al.*, 1990). The objective of the present work is to analyse further the group of tumours in which such alterations were found, from both clinical and pathological approaches, to evaluate the prognostic significance of MHC class I antigens in patients with laryngeal carcinoma.

### Materials and methods

### *Patients*

Sixty patien s with squamous cell carcinoma of the larynx were included in the study. None of them received radiotherapy or chemotherapy prior to surgery. All were male. Age ranged from 44 to 75 years (average 58.68). Tumours were classified as originating from the supraglottic region (33; 55%), glottis (18; 30%), subglottis (2; 3.3%) and pyriform sinus (3; 5%). Four cases were considered transglottic (6.7%).

Surgical techniques consisted in cordectomy (four cases), frontolateral laryngectomy with epiglottoplasty as first described by Sedlàcek and popularised by Tucker (1976) (two cases), partial horizontal supraglottic laryngectomy (12 cases) and total laryngectomy (42 cases). Twenty-two patients underwent ipsilateral and 18 bilateral functional neck dissection. Radical ipsilateral neck dissection was performed in three patients, and a bilateral procedure in four. Follow-up ranged from 12 to 48 months. At present, ten patients have died of laryngeal cancer, and one is alive with disease. Two cases were lost to follow-up. In each case we recorded the location and diameter of the primary tumour, T and N pathological staging (Kleinsasser, 1987), number of lymph nodes from the neck dissection and number of lymph node metastases, pathological staging, blood group and Rh of the patient, age, smoking and alcohol consumption, first symptom-diagnosis interval and follow-up (in months).

### *Pathological analysis*

Tumours were classified into three grades (UICC modified Broders' system (Wahi, 1972)) and all were scored according to Glanz's (1984) and Jakobsson's (Jakobsson *et al.*, 1973) grading systems for squamous cancer. In addition, two modifications to the former were included (Crissman *et al.* (1984) and the authors' modification). The histologic grade of malignancy was based upon the tumour cell population (structure, differentiation, nuclear polymorphism, mitoses) and tumour–host relationship (mode of invasion, stage of invasion, vascular invasion, cellular response). Histological analyses were performed without any knowledge of the clinical stage, treatment, or further course of the disease.

### *Immunohistochemical analysis*

The immunohistochemical technique and monoclonal antibodies used were described in a previous publication (Esteban *et al.*, 1989). A tumour was classified as negative when no staining was detected in any of ten randomly chosen microscopic fields, and as positive when all tumour cells were stained in ten fields. In our series, no specimens showed heterogeneous pattern of staining for the HLA-ABC antigens.

### *Statistical analysis*

The mean and standard deviation of each pathological and clinical parameter were computed. HLA (+) tumours and HLA (−) tumours were compared to by a *t* test. Statistical correlations were calculated using the BMDP package from UCLA (1985 version).

Correspondence: F. Garrido.
Received 24 May 1990; and in revised form 25 July 1990.

**Figure 1** A HLA class I positive tumour, presenting homogeneous staining with the W6/32 monoclonal antibody against the heavy chain of class I antigens. APAAP technique (× 137.5).

**Figure 2** HLA class I negative laryngeal carcinoma. Positivity was limited to the stromal cells and tumour infiltrating leukocytes. GRH1 monoclonal antibody against $\beta_2$-microglobulin. APAAP technique (× 137.5).

**Figure 3** Relationship between HLA class I expression and degree of differentiation in our series. $P = 0.001$.

### Results

Normal laryngeal mucosa was always positive for HLA-ABC antigens. Losses of these antigens were always related to malignancy. In Figure 1 we present an example of well differentiated squamous cell carcinoma of the larynx, showing homogeneous staining for the W6/32 monoclonal antibody against the heavy chain of HLA class I antigens. Forty-one carcinomas were considered positive for class I antigens.

We found 13 total losses of HLA-ABC antigens, as revealed by the W6/32 and GRH1 monoclonal antibodies (Figure 2), five selective losses of HLA-A (anti-HLA-A MAb) and one of HLA-B antigens (anti-HLA-B). Our analysis of class I expression in tumours and autologous lymph node metastases (15 cases) detected four cases presenting divergencies: in two cases the primary tumour was negative and the metastases positive, and in one case the metastases were negative while the tumour was positive. The fourth tumour was negative, but the metastases presented selective loss of HLA-B antigens. We should point out that in two cases selective loss of HLA-A antigens was found not only in the primary tumour but also in the metastases.

### *Pathological parameters*

When comparing HLA-ABC (−) tumours with the HLA-ABC (+) group, we found a clear relationship between class I expression and degree of differentiation. HLA-ABC (−) tumours presented a worse differentiation, based on Broders' (Figure 3), Jakobsson's or Glanz's grading systems (Table I). The study of the pathological parameters between groups also yielded differences (Table II).

### *Clinical parameters*

The clinical parameters significantly associated with the loss of class I antigens are shown in Table III. We excluded tumours of the pyriform sinus from our analysis, as they present a different biological behaviour, and most series consider them separately. The comparison of the mean interval between first symptom and diagnosis also yielded significant differences.

The relationship between HLA-ABC losses (total and selective losses) and metastatic potential is shown in Figure 4. The $P$ value was 0.1771. In this analysis we excluded both pyriform sinus carcinomas and verrucous carcinomas in order to work with a homogeneous group.

Most class I negative tumours were classified as advanced stages (Figure 5). We found a close relationship between class I expression and staging of the cases, most of the negative class I tumours being stage IV (9 out of 12). As there was no significant association between metastatic potential and class I losses, it seems that HLA class I negative tumours were bigger and grew deeper than those in the positive group. At present, ten patients had died of disease, and six of them had tumours presenting total or selective loss of HLA-ABC antigens.

### Discussion

Because of the central role of class I and II molecules in the

**Table I** Comparison of HLA (+) and (−) tumours: grading systems

| *System* | *Cases (+)*[b] | *s.d.*[a] | *Cases (−)*[c] | *s.d.* | *Significance* |
|---|---|---|---|---|---|
| Broders | 1.634 | 0.79 | 2.462 | 0.66 | $P = 0.001$ |
| Jakobsson | 17.51 | 4.88 | 23.08 | 3.90 | $P = 0.0003$ |
| Jakob. mod.[d] | 13.73 | 4.64 | 19.08 | 3.90 | $P = 0.0004$ |
| Jakob. mod.[e] | 15.59 | 4.25 | 20.19 | 3.25 | $P = 0.0003$ |
| Glanz | 5.02 | 2.20 | 6.92 | 1.55 | $P = 0.0018$ |

[a]Standard deviation. [b]HLA (+) tumours (41 cases). [c]HLA (−) tumours (13 cases). [d]Crissman *et al.* (1980). [e]Authors' modification including 'overall cellular differentiation'.

**Table II** Comparison of HLA (+) and (−) tumours: pathological parameters

| *Parameter* | *Tumours (+)*[a] | *Tumours (−)*[b] | *Significance* |
|---|---|---|---|
| *Jakobsson*[c] | | | |
| Differentiation[d] | 1.872 | 2.769 | $P = 0.0049$ |
| Nuclear atypia | 1.897 | 2.846 | $P = 0.0016$ |
| Number of mitosis | 2.026 | 2.692 | $P = 0.0163$ |
| Structure | 1.744 | 1.899 | $P = 0.5525$* |
| Pattern of invasion | 1.699 | 2.000 | $P = 0.4484$* |
| Vascular invasion | 2.154 | 3.538 | $P = 0.0053$ |
| Lymphoid response | 1.872 | 2.308 | $P = 0.0600$* |
| Stage of invasion | 3.769 | 4.000 | $P = 0.1441$* |
| Differentiation[e] | 1.897 | 2.885 | $P = 0.0011$ |
| *Glanz* | | | |
| Differentiation | 1.667 | 2.462 | $P = 0.0022$ |
| Structure and margins | 1.385 | 1.538 | $P = 0.3751$* |
| Vascular/perineural infiltration | 1.000 | 1.615 | $P = 0.0154$ |
| Infiltrate | 0.846 | 1.385 | $P = 0.0273$ |

*No significance was found. [a]HLA (+) tumours. [b]HLA (−) tumours. [c]Parameters included in Jakobsson's grading system and its modifications. [d]Differentiation of the tumour (keratin formation). [e]Cellular differentiation.

**Figure 4** Relationship between HLA class I expression and metastatic potential of the tumours.

**Figure 5** Relationship between HLA class I expression and staging of the tumours.

immune system, it is not surprising that alterations in their expression could affect the immunosurveillance against tumours. During the cytotoxic-T-lymphocyte-mediated response, the neoplastic antigen associated with class I products interact with the T cell receptor (TCR), thus changes in the expression of histocompatibility antigens may affect the immune response against malignant neoplasms, their growth rate and metastatic potential. The expression of these molecules may therefore be one of the factors responsible of oncogenicity, due to their role as restriction elements in T cell recognition (Zinkernagel & Doherty, 1979).

Although our studies do not elucidate the molecular basis of the defect in expression of class I antigens, regulatory mechanisms, rather than genetic rearrangements, are likely to be involved in these phenomena, as shown by the results of the southern blotting analyses in our series (Esteban *et al.*, 1989). In fact, in many human tumours with undetectable levels of class I antigen expression, HLA expression has been induced by exposing the neoplastic cells to τ-interferon (Ruiz-Cabello *et al.*, 1988, 1989). Further evidence supporting the hypothesis that regulatory mechanisms are involved in the control of the expression of MHC products comes from oncogene studies. The amplification of c-*myc* has been shown to correlate with low levels of class I molecules (Doyle *et al.*, 1985), and other studies in neuroblastoma (Lamson *et al.*, 1983) and melanoma cell lines (Versteeg *et al.*, 1988) have also found an association between c-*myc* and N-*myc* amplification and a decrease in class I molecule expression. These authors claim that repression of the expression may be a common feature in oncogene-mediated malignant transformation.

Our comparative analysis of tumours with total loss of MHC class I antigen expression with tumours showing levels of expression considered normal suggest a number of conclusions. To assess the malignancy of these tumours, because of the short follow-up period (12–48 months), we used the criteria of Jakobsson *et al.* (1973) with two modifications (Crissman *et al.* (1984) and our own modification) and criteria established by Glanz (1984). As described in Table II,

**Table III** Clinicopathological parameters: HLA class I (+) and (−) tumours

| *Parameter*[a] | *Cases (+)*[b] | *s.d.*[e] | *Cases (−)*[c] | *s.d.* | *Significance* |
|---|---|---|---|---|---|
| T stage | 2.872 | 0.80 | 3.538 | 0.51 | $P = 0.0014$ |
| First symptom–diagnostic interval[d] | 15.71 | 23.73 | 4.583 | 4.14 | $P = 0.0091$ |
| Pathological staging | 2.949 | 0.85 | 3.769 | 0.43 | $P = 0.0001$ |

[a]Mean of values for each parameter. Tumours of the pyriform fossa were excluded. [b]Group of HLA (+) tumours. [c]Class I negative tumours. [d]In months. [e]Standard deviation.

the loss of HLA class I antigen expression is associated with tumours of worse prognosis according to the grading systems employed. When analysing the different pathological parameters used and their significance values, lower *P* values were obtained in association with nuclear atypia, vascular invasion and overall cellular differentiation (Table II). The strong association with vascular invasion was somewhat amazing, as tumours with altered class I expression have not been significantly associated with the presence of metastases (Figure 4). We should however point out that vascular invasion itself has been linked with an ominous prognosis (Poleksic & Kalwaic, 1978). Tumour cell lysis mediated by NK cells could be an explanation of this finding.

Another apparent discrepancy was that tumours with altered class I expression were not significantly associated with advanced stages of histopathological invasion (Table II), whereas the association was evident with advanced clinicopathological invasion ($P = 0.0001$; Table III). This may be derived from the grading systems used, as most tumours were classified as showing 'deep infiltration', whereas they differed clearly enough to be categorised as T3 or T4 when studying the surgical specimens.

On the other hand, we have observed that the loss of class I antigens is strongly associated with the degree of differentiation (Figure 3), a phenomenon also noted in other carcinomas, i.e. colon carcinomas (Momburg *et al.*, 1986). There was a clear relationship between loss of class I antigens and the different grading systems of malignancy employed, as described in Table I. When analysing the different parameters which are considered to reflect the differentiation of the tumour cell, the strongest association for class I tumours was found with the parameter that we called 'overall cellular differentiation; ($P = 0.0011$). A less marked but still high significant association was noted for cytoplasmic differentiation (keratin formation) (Table II).

There was a strong relationship between the absence or selective loss of class I molecules and the T stage (TNM classification), but not with maximum diameter of the neoplasm, suggesting that the main factor was degree of deep invasion of the tumour rather than superficial spreading. This phenomenon has also been observed in malignant melanoma (Bröcker *et al.*, 1985) and may be explained in terms of 'tumour' progression', i.e. the natural history of spontaneous tumours being a multistep process in which new clones replace their precursors as a result of selection (Cairns, 1975; Klein & Klein, 1985). The loss of class I antigen expression associated with advanced T stages may result from phenotypical changes which benefit the neoplastic cell growth. In this way, the classical studies of the different anatomical and biochemical laryngeal barriers are of significance (Tucker, 1976; Kirchner & Carter, 1987). Moreover, in contrast to the large number of studies relating the degree of differentiation to metastatic potential (see for review in Glanz, 1984), only a limited number of publications have linked larger tumours with higher rates of metastasis (McGavran *et al.*, 1961; Kirchner *et al.*, 1974; Pera *et al.*, 1986). The analysis of tumours using the different grading systems of malignancy seems to point toward a gradient from positive tumours with lower scores and better prognosis, to HLA-ABC negative tumours, being a intermediate group the neoplasms with selective losses of HLA-A or HLA-B antigens (data not shown). It is interesting to remark the other clinicopathological parameters associated with alterations in class I expression. Duration of symptoms is an indirect measure of tumour aggressiveness, or at least of the rate of neoplastic growth. Notably, class I negative tumours also exhibited a short first symptom–diagnosis interval (Table III) with a significant difference between negative (mean 4.5 months) and positive tumours (mean 15.7 months, $P = 0.0091$). Thus, class I negative tumours seem to show a highly aggressive histopathological profile, as shown by the significance found in all the grading systems used, as well as on the basis of clinical criteria, i.e. association with advanced T stage and short first symptom–diagnosis interval, and worse prognosis as reflected by the patient's status.

In conclusion, the association between loss of HLA-ABC expression and a number of parameters related to tumour aggressiveness supports the hypothesis that the study of class I expression in laryngeal cancer may be considered of clinical value. A negative result for class I antigen expression may indicate deep infiltration by the tumour, which could be difficult to detect before surgery has been performed. Further investigations will be needed to ascertain the role of HLA antigens in the immunobiology of laryngeal squamous cell carcinoma, being follow-up studies necessary to evaluate the prognostic significance of the alterations in class I antigen expression.

### References

BRÖCKER, E.B., SUTER, L., BRUGGEN, J., RUITER, D.J., MACHER, E. & SORG, C. (1985). Phenotypic dynamics of tumor progression in human malignant melanoma. *Int. J. Cancer*, **36**, 29.

CAIRNS, J. (1975). Mutation selection and the natural history of cancer. *Nature*, **255**, 197.

CRISSMAN, J.D., LIU, M.Y., GLUCKMAN, J.L. & CUMMINGS, G. (1984). Prognostic value of histopathologic parameters in squamous cell carcinoma of the oropharynx. *Cancer*, **54**, 2995.

DOYLE, A., MARTIN, J., FUNE, K. & 8 others (1985). Markedly decreased expression of class I histocompatibility antigens, proteins and mRNA in human small-cell lung cancer. *J. Exp. Med.*, **161**, 1135.

ESTEBAN F., CONCHA, A., HUELIN, C. & 4 others (1989). Histocompatibility antigens in primary and metastatic squamous cell carcinoma of the larynx. *Int. J. Cancer*, **43**, 436.

ESTEBAN, F., CONCHA, A., PEREZ-AYALA, M., SANCHEZ-ROZAS, J.A., RUIZ-CABELLO, F. & GARRIDO, F. (1990). DR expression is associated with an excellent prognosis in squamous cell carcinoma of the larynx. *Clin. Exp. Meth.*, **8**, 319.

FENTENSTEIN, H. & GARRIDO, F. (1986). MHC antigens and malignancy. *Nature*, **322**, 502.

GARRIDO, F. (1987). The biological implications of the abnormal expression of histocompatibility antigens on murine and human tumors. In *H-2 Antigens*, David, C.S. (ed.) p. 623. Plenum Publishing: New York.

GLANZ, H. (1984). Carcinoma of the larynx. Growth, p-classification and grading of squamous cell carcinoma of the vocal cords. *Adv. Oto-Rhino-Laryngol.*, **32**, 1.

JAKOBSSON, P.A., ÆNNEROTH, C.M., KILLANDER, P.D., MOBERGER, G. & MÅRTENSSON, B. (1973). Histologic classification and grading of malignancy in carcinoma of the larynx. *Acta Radiol. Ther. Phys. Biol.*, **12**, 1.

KLEIN, G. & KLEIN, E. (1985). Evolution of tumours and the impact of molecular oncology. *Nature*, **315**, 190.

KLEINSASSER, O. (1987). *Tumours of the Larynx and Hypopharynx.* Thieme Medica: New York.

KIRCHNER, J.A., CORNOG, J.L. & HOLMES, R.E. (1974). Transglottic cancer. Its growth and spread within the larynx. *Arch. Otolaryngol.*, **94**, 247.

KIRCHNER, J.A. & CARTER, D. (1987). Intralaryngeal barriers to the spread of cancer. *Acta Otolaryngol.*, **103**, 503.

LAMPSON, L.A., FISCHER, C.A. & WHELAN, J.P. (1983). Striking paucity of HLA-A,B,C and $\beta_2$-microglobulin in human neuroblastoma cell lines. *J. Immunol.*, **130**, 2471.

LOPEZ-NEVOT, M.A., GARCIA, E., PAREJA, E. & 5 others (1986). Differential expression of HLA Class I and II antigens in primary and metastatic melanomas. *J. Immunogenet.*, **13**, 219.

LOPEZ-NEVOT, M.A., ESTEBAN, F., FERRON, A. & 6 others (1989). HLA class I gene expression on human primary tumors and autologous metastases: demonstration of selective losses of HLA antigens on colorectal, gastric and laryngeal carcinomas. *Br. J. Cancer*, **59**, 221.

McGAVRAN, M.H., BAUER, W.C. & OGURA, J.H. (1961). The incidence of cervical lymph node metastases from epidermoid carcinoma of the larynx and their relationship to certain characteristics of the primary tumour. *Cancer*, **14**, 55.

MOMBURG, F., MÖLLER, P., MOLDENHAUER, G. & HÄMMERLING, G.J. (1986). Loss of HLA-A,B,C in colorectal carcinoma is related to the degree of dedifferentiation. *J. Immunogenet.*, **13**, 195.

PERA, E., MORENO, A. & GALINDO, L. (1986). Prognostic factors in laryngeal carcinoma. A multifactorial study of 416 cases. *Cancer*, **58**, 928.

POLEKSIK, S. & KALWAIC, H.J. (1978). Prognostic value of vascular invasion in squamous cell carcinoma of the head and neck. *Plast. Reconstr. Surg.*, **61**, 234.

RUIZ-CABELLO, F., LOPEZ-NEVOT, M.A. & GARRIDO, F. (1988). MHC Class I and II gene expression on human tumors. In *Cancer Metastasis*, Prodi, G., Liotta, L.A., Lollini, P.-L., Garbisa, S., Gorini, S. & Hellman, K. (eds) p. 119. Plenum Publishing: New York.

RUIZ-CABELLO, F., LOPEZ-NEVOT, M.A., GUTIERREZ, J. & 8 others (1989). Phenotypic expression of histocompatibility antigens in human primary tumors and metastases. *Clin. Exp. Meth.*, **7**, 213.

TOWNSEND, A.R.M., GOTCH, F.M. & DAVEY, J. (1985). Cytotoxic T cells recognize fragments of the influenza nucleoprotein. *Cell*, **42**, 457.

THORSBY, E. (1982). Involvement of HLA cell membrane molecules in T cell immune responses: Immunological and clinical significance. In *HLA Typing: Methodology and Clinical Aspects, Vol. 2*, Ferrone, S. & Solheim, B.G. (eds) p. 151. CRC Press: Boca Raton.

TUCKER, G.F. (1976). The anatomy of layngeal cancer. In *Workshops of the Centennial Conference on Laryngeal Cancer*, Alberti, P.W. & Bryce, D.P. (eds) p. 11. Appleton-Century-Crofts: New York.

VERSTEEG, R., NOORDERMEER, I.A., KRUSE-WOLTERS, M., RUITER, D.J. & SCHRIER, P.I. (1988). C-myc down-regulates class I expression in human melanomas. *EMBO J.*, **7**, 1023.

WAHI, P.N. (1972). *Histological Typing of Oral and Oropharyngeal Tumors*. WHO: Geneva.

ZINKERNAGEL, R.M. & DOHERTY, P.C. (1979). MHC-restricted cytotoxic cells. Studies of the biological role of polymorphic major transplantation antigens determining T-cells' restriction, function and responsiveness. *Adv. Immunol.*, **27**, 51.